# Fabrication and evaluation of bilateral Helmholtz radiofrequency coil for thermo‐stable breast image with reduced artifacts

**DOI:** 10.1002/acm2.13483

**Published:** 2021-12-01

**Authors:** Young Han Lee, Kyu‐Ho Song, Jaemoon Yang, Won Jun Kang, Keum Sil Lee, Min Jung Kim, Eun‐Kyung Kim, Dan Heo, Bo‐Young Choe, Jin‐Suck Suh

**Affiliations:** ^1^ Department of Radiology Severance Hospital Research Institute of Radiological Science Yonsei University College of Medicine Seoul Republic of Korea; ^2^ Department of Radiology Washington University School of Medicine St. Louis Missouri USA; ^3^ Department of Nuclear Medicine Severance Hospital Yonsei University College of Medicine Seoul Republic of Korea; ^4^ Department of Radiology Stanford University School of Medicine Stanford California USA; ^5^ Department of Biomedical Engineering College of Medicine and Research Institute of Biomedical Engineering The Catholic University of Korea Seoul Republic of Korea

**Keywords:** breast imaging, magnetic resonance imaging, metallic artifact, positron emission tomography, radiofrequency coil

## Abstract

**Purpose:**

The positron emission tomography (PET)‐magnetic resonance (MR) system is a newly emerging technique that yields hybrid images with high‐resolution anatomical and metabolic information. With PET‐MR imaging, a definitive diagnosis of breast abnormalities will be possible with high spatial accuracy and images will be acquired for the optimal fusion of anatomic locations. Therefore, we propose a PET‐compatible two‐channel breast MR coil with minimal disturbance to image acquisition which can be used for simultaneous PET‐MR imaging in patients with breast cancer.

**Materials and Methods:**

For coil design and construction, the conductor loops of the Helmholtz coil were tuned, matched, and subdivided with nonmagnetic components. Element values were optimized with an electromagnetic field simulation. Images were acquired on a GE 600 PET‐computed tomography (CT) and GE 3.0 T MR system. For this study, we used the T1‐weighted image (volunteer; repetition time (TR), 694 ms; echo time (TE), 9.6 ms) and T2‐weighted image (phantom; TR, 8742 ms; TE, 104 ms) with the fast spin‐echo sequence.

**Results:**

The results of measuring image factors with the proposed radiofrequency (RF) coil and standard conventional RF coil were as follows: signal‐to‐noise ratio (breast; 207.7 vs. 175.2), percent image uniformity (phantom; 89.22%–91.27% vs. 94.63%–94.77%), and Hounsfield units (phantom; ‐4.51 vs. 2.38).

**Conclusions:**

Our study focused on the feasibility of proposed two‐channel Helmholtz loops (by minimizing metallic components and soldering) for PET‐MR imaging and found the comparable image quality to the standard conventional coil. We believe our work will help significantly to improve image quality with the development of a less metallic breast MR coil.

## 1 INTRODUCTION

Recently, the use of positron emission tomography (PET)‐magnetic resonance (MR) hybrid images which combine the high resolution of anatomical imaging to reveal internal structures (e.g., soft tissue, skin, and bone) is expected to improve the diagnostic performance of biomarker detection and to provide more diagnostic information on malignancies with radiotracer metabolism.^1^ In addition, PET‐MR hybrid images (called simultaneous imaging) can be potentially used for various diagnostic purposes in oncology, cardiology, and neurology with a real‐time recording of morphologic, physiologic, functional, and metabolic study.^2^ And hybrid imaging certainly offers advantages in terms of patient management and time savings by avoiding patient relocation with co‐registration and localization of anatomy and lesions.^3^ Research has been actively conducted on the PET‐MR scanner and its application to solid malignant tumors in several organs such as the lung, prostate, and cervix.^4^, ^5^, ^6^, ^7^ However, even though breast cancer is one of the most common cancers in women worldwide, it is difficult to obtain PET‐MR hybrid images of the breast because of different positions needed for the two individual techniques, with the prone position being incorporated in MR and the supine position in PET.

Breast MR imaging requires an enhanced radiofrequency (RF) breast coil to obtain high‐resolution and high‐contrast images of the breast,^8^ and PET‐MR imaging requires high spatial accuracy and patient‐comfort for optimal MR positioning. However, with the current conventional dedicated breast coil, there are some limitations when the breast coil lies within the field‐of‐view (FOV) of computed tomography (CT) or PET scan. The metallic components of the breast coil with numerous leads and solders disturb photon transmission and image acquisition during the PET scan despite attenuation correction (AC). Also, these electronic components of the RF circuits can reduce image quality while causing image artifacts.^9^ Therefore, the presence of metallic components affects data quantification in PET.^10^ Metallic components within the FOV of a PET scan can affect the photon starvation, beam hardening, and scattering in patients who underwent PET‐CT scan, leading to image quality degradation, adverse artifacts, or inaccurate standard uptake values (SUVs) calculation. Even tiny metallic components such as implanted leads in a cardioverter‐defibrillator or pacemaker can cause metallic artifacts.^11^ A software approach to metallic artifact reduction (MAR) based on the prior image has been introduced^12^ and applied in patients with metallic implants.^13^ However, there were debates about the under‐ and over‐estimation of fluorodeoxyglucose (FDG) activity concentration with MAR algorithms, especially in the most artifact‐affected areas around metal prostheses. Magnetic coil design consideration can be an alternative hardware approach. Therefore, the presence of metallic components needs to be considered when designing a breast coil.^10^, ^14^, ^15^ If the interference to image acquisition caused by the dedicated breast RF coil could be decreased or removed,^16^ better anatomical and functional information could be obtained from single modality or hybrid images for breast imaging and the MR and PET scanner could emerge as a more accurate diagnostic tool for breast lesions.^17^, ^18^ In this paper, we describe a two‐channel Helmholtz breast coil for 3.0 T MR that consists of a butterfly bilateral shape placed on a closed acrylic plate. Also, we incorporated two‐channel breast coils (one channel on each side) to actualize this first feasibility approach. The aims of this study were to modify the conventional breast coil for a PET‐MR scanner, to minimize interference to data acquisition during the PET scan, and to improve image quality based on CT images with a comparable signal‐to‐noise ratio (SNR) during the MR examination, in comparison with PET‐CT images obtained with the conventional breast MR coil.

## 2 MATERIALS AND METHODS

### 2.1 RF coil design for improved image performance

The proposed novel RF coil consisted of two‐channel Helmholtz loops by minimizing metallic components and soldering. Beam‐hardening and photon starvation artifacts due to the metallic components of a coil can be disruptive in PET‐CT imaging. By reducing the diameter and size of the coil, we can minimize the potential of metallic artifacts. For small FOV acquisition, the coil was designed to fit into a coil frame used in clinical practice (Invivo Corp, Gainesville, FL, USA). A simple strategy was chosen to focus on breast tissue by reducing the imaging FOV as close as possible to include breast tissue while avoiding metallic artifacts.

**FIGURE 1:**
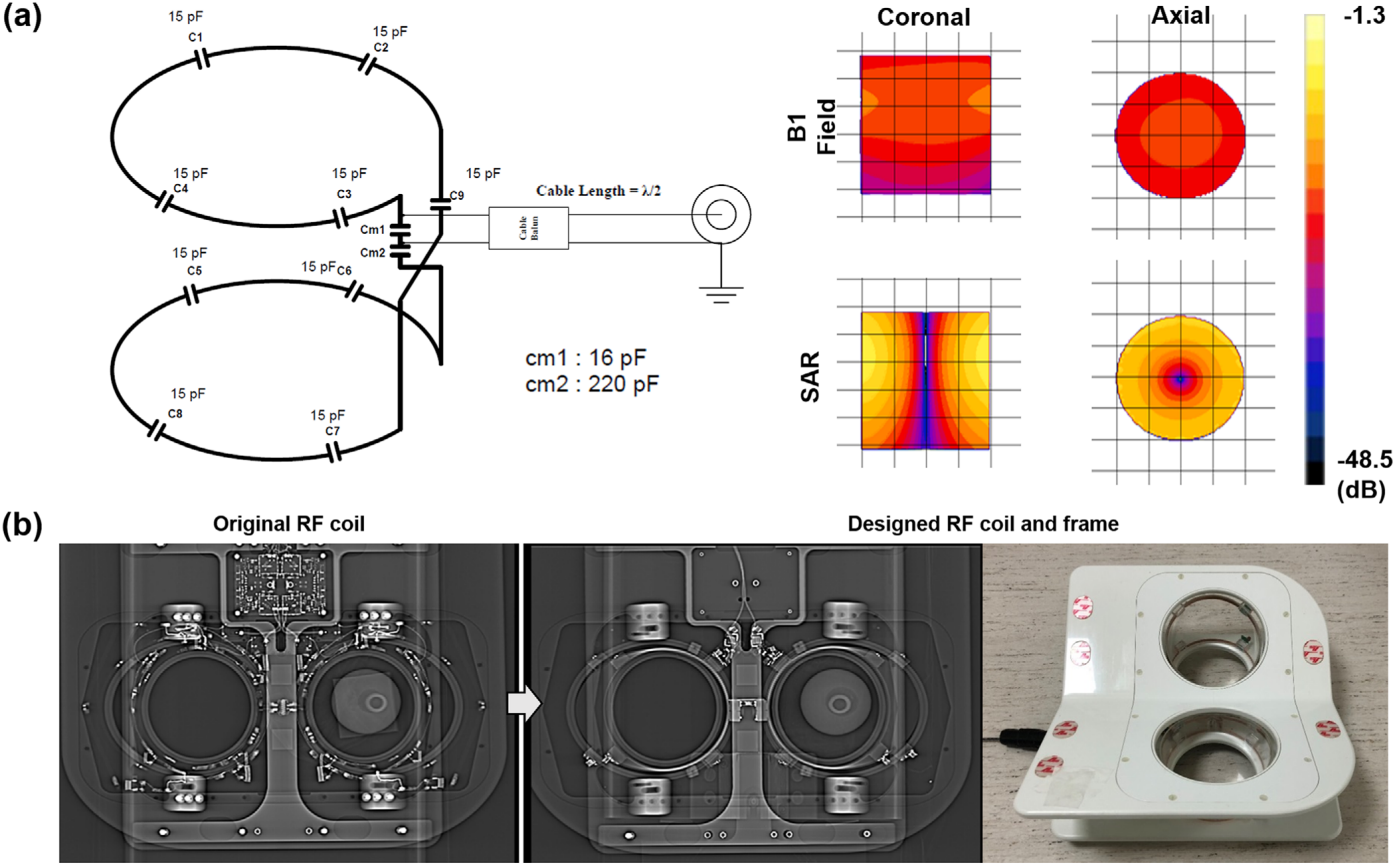
The designed bilateral breast coil. (a) Schematic drawing of the designed bilateral breast coil. Simulation test: B1 field was normalized to 5 × 10^−13^ V/m^2^ as 0.0 dB, and specific absorption rate (SAR) was normalized to 1.0 × 10^−14^ W/kg as 0.0 dB. (b) Comparison images of one of commercially available breast radiofrequency (RF) coils and designed RF breast coil. Compared with the scout (X‐ray) image prior to modifications, the majority of the metal components were minimized after modification

For electromagnetic simulation, a SEMCAD X (Ver. 14.2.1 Schmid & Partner Engineering AG, Zurich, Switzerland) as three‐dimensional (3D) full‐wave electromagnetic and thermal simulation platform based on the finite‐difference time‐domain (FDTD) and finite element method (FEM) was employed to design the coil geometry and numerical loading phantom. The intrinsic B1 field distribution in the numerical phantom (and the buffers) with a cylindrical shape full of body fluid with relative permittivity (*ε*_r_ = 69.062), electric conductivity (*σ* = 1.5053 S/m), and loss tangent (tan *δ* = 3.0851) was investigated. The numerical phantom was dimensioned with the loading phantom that was used for the experimental *Q*‐factor measurements and MR image scanning (Figure 1a).

With the bilateral breast coil design used in the simulation, an in‐house breast coil array was constructed. Each loop element was set to 13 (diameter) × 11 (max height) × 6 (min height) cm^3^ for appropriate depth penetration (Figure 1a). The cable baluns (thickness (diameter), 3 cm; length, 8 cm) were surrounded by foam sleeves to minimize their interaction with the environment. Bench measurements for coil tuning and matching were performed on a Hewlett‐Packard 8753D Network Analyzer (Hewlett Packard, Palo Alto, CA, USA), and the coils were tuned to 127.74 MHz. Inter‐mutual coupling between the left and right coils was resolved with decoupling transformer circuits. *Q*‐factors with the loading phantom were measured with a dual sniffer probe. For the plastic frame of the breast coil, a coil frame was modified. All of the metal screws in the frame were replaced with plastic screws and ceramic capacitors, previously embedded coils were removed, and the remaining metallic components in the local FOV area were removed and rearranged in an area out of the local FOV on comparison radiographs (Figure 1b).

For MR scanning, the coil was connected to a coil interface box (3.0 T 1H gateway; Clinical MR Solutions, Brookfield, WI, USA). The coil interface box included eight low‐noise amplifiers (LNA) tuned at 3.0 T (127.7 MHz). The LNA had a 25 dB gain, 0.4 dBm noise figure, and 1 ± 0.5 Ω input impedance. The specifications for LNA were nominal values. The decoupling/coupling driver voltages (‐5 V/3 V) derived from the coil interface box supplied the coils separately.

To compare the designed coil with the commercially available bilateral eight‐channel breast coil (equivalent to the conventional coil) used in routine clinical practice, artifacts, efficacy, and image quality were evaluated for both coils.

### 2.2 Acquisition of imaging

The PET‐CT scans were obtained on a Discovery 600 PET‐CT (GE Healthcare, Waukesha, WI, USA). ^18^F‐FDG (0.5 mCi) was mixed with normal saline in a plastic bottle, 7 cm in diameter and 9 cm in height, which was used as a phantom to evaluate image artifacts. CT and PET images of the phantom with FDG were obtained with each coil for comparison. CT scans were performed prior to obtaining PET images with the following parameters: voltage and current, 120 kVp and 60 mA; exposure time, 5074 ms; data collection diameter, 500 mm; distance source to detector, 949.075 mm; distance source to object, 541 mm; standard body filter; slice thickness, 3.75 mm; helical mode; spiral pitch factor, 0.9375; single collimation width, 1.25; total collimation width, 20; CT dose index (CTDI) vol, 2.41 mGy. These CT images were converted to a 511 keV photon attenuation map and used for PET AC. PET images were reconstructed using a standard Ordered Subset Expectation Maximization (OSEM) 3D‐iterative algorithm with *n* iterations and *nn* subsets.

All MR scans were performed on a 3.0 T clinical scanner (Discovery MR750, GE Healthcare). The scan parameters were as follows: (1) fast spin‐echo (FSE) T2‐weighted image for the loading phantom; repetition time (TR), 8742 ms; echo time (TE), 104 ms; flip angle (FA), 111°; matrix size, 416 × 256; FOV, 320 mm × 320 mm; slice thickness, 3 mm; slice gap, 0 mm; echo train length (ETL), 20; number of excitations (NEX), 1; scan time, 1 min 57 s; (2) FSE T2‐weighted image for the temperature safety evaluation; TR, 8742 ms; TE, 99.792 ms; FA, 111°; matrix size, 512 × 512; FOV, 320 mm × 320 mm; slice thickness, 3 mm; slice gap, 0 mm; NEX, 16; ETL, 32; scan time, 31 min 38 s.

**FIGURE 2:**
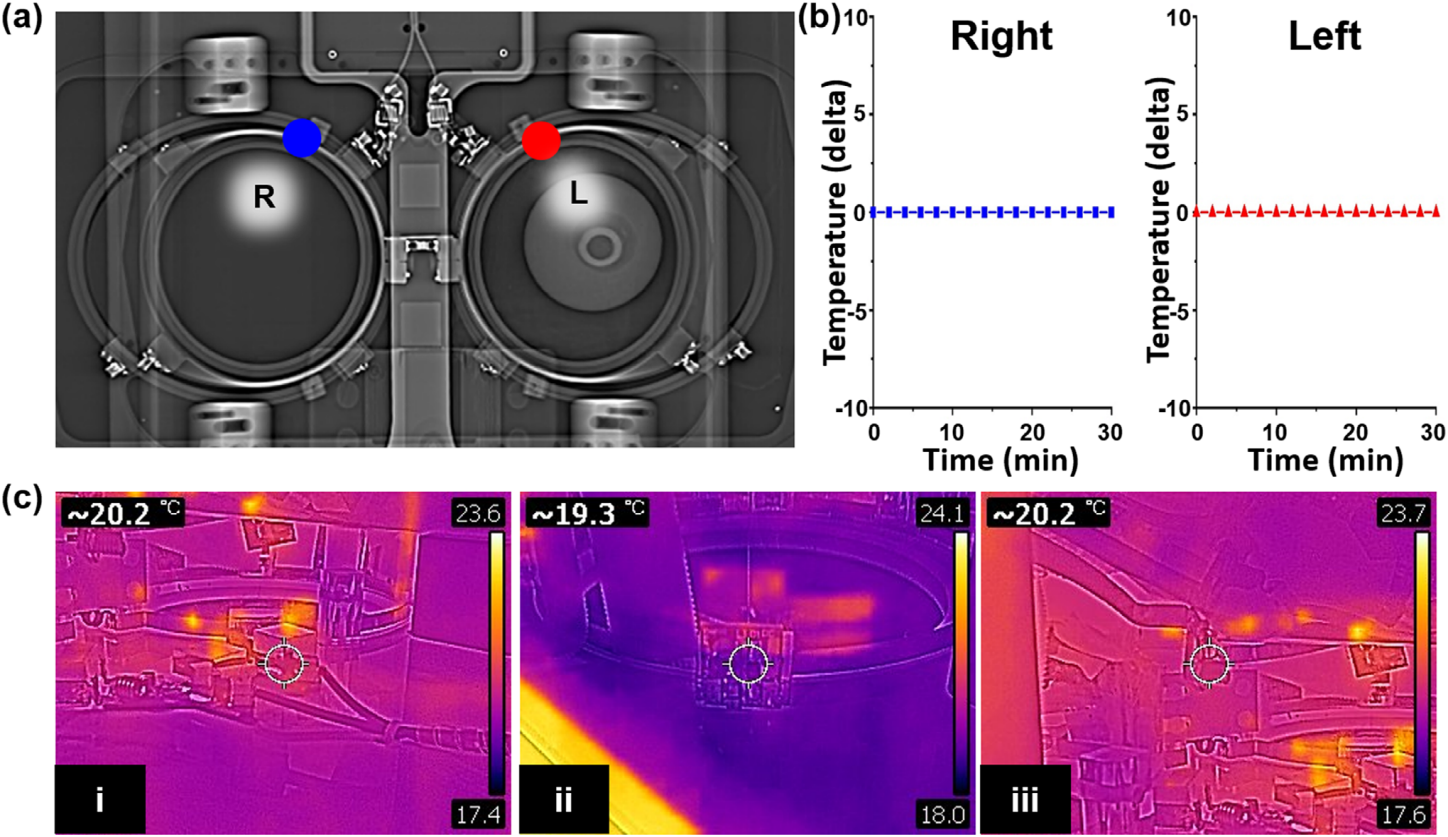
Temperature test of the designed coil. (a) Locations of temperature sensor near the coil circuit. (b) Time–temperature curve shows less than 1° of change during a 30‐min continuous scan. (c) Various views of temperature were shown for infrared (forward looking infrared, FLIR) C2 imaging camera. Several temperature measurements show not significantly temperature increasement with the FLIR C2 imaging camera

To evaluate safety with respect to temperature, temperature changes occurring during scanning were directly monitored throughout the MR examination for a maximum of 33 min simultaneously using an MR‐compatible optic temperature probe (AccuSens; opSens, Quebec, Canada) within the coil. The optical probes had temperature sensors near their ends, and optical probes were located at the inner wall of the coil at the R position for the right breast and L position for the left breast (Figure 2a,b). The temperature differences in the designed coil after the MR examination were recorded by an infrared (IR) temperature imaging camera (forward looking infrared (FLIR) C2, FLIR Systems, Inc., Wilsonville, OR, USA) with 320 × 240 pixels and 4800‐pixel image resolution in a 2D measurement (Figure 2c).

### 2.3 Image quality based on PET‐CT

To evaluate image quality and artifacts due to metal components on the PET scan, we used the CT image, which is typically considered as an appropriate reference for the evaluation of artifacts from a metallic component.^10^ Image quality was evaluated visually for PET and CT images and quantitatively for CT images. Image homogeneity was also measured using Hounsfield units (HU) from the selected region of interest (ROI) that encompassed 75% of the bottle phantom. The average HU and standard deviation were calculated for the phantom only and for the phantom in the designed coil and in the conventional coil. The noise was semi‐quantified by the standard deviation of HU in phantoms with or without (w/o) the coil. The maximal and mean SUVs of attenuation‐corrected PET images were measured at the center slice of the phantom and the peripheral slice of the phantom image 1 cm from the center for each coil.

### 2.4 Signal‐to‐noise ratio measurements

Images were acquired to measure the SNR (*S*_image_ /SD_noise_) and percent image uniformity (%PIU = [1 – {(*S*_max_ – *S*_min_)/(*S*_max_ + *S*_min_)}] × 100) for the intensity uniformity with the acceptance value (≥82.0% at 3.0 T). The loading phantom, a bottle with a diameter of 115 mm and height of 240 mm (Invivo, Pewaukee, WI, USA), filled with 2.0 ± 0.05 g/L CuSO_4_·5H_2_O, 4.5 ± 0.05 g/L NaCl, and 1.89 L distilled water, was placed vertically at the center of the RF coil. The axial slice covering the middle of the loading phantom was used to calculate the SNR and %PIU within a local FOV. The mean signal (*S*_image_) was defined as the average pixel intensity in an approximately 150 mm^2^‐sized round ROI covering the loading phantom. The standard deviation (SD_noise_) of noise was measured in background regions (approximately 20 mm^2^‐sized oval ROIs) drawn over an area of no signal (i.e., air) and averaged separately. The maximum pixel intensity (*S*_max_) and minimum pixel intensity (*S*_min_) of the signal were obtained from pixels within an ROI that encompassed 75% of the loading phantom.^19^

### 2.5 Image quality based on clinical breast MR images in a volunteer

Clinical breast MR images for the volunteer scan were acquired using the following scan parameters: (1) T1‐weighted image with spectral fat suppression; TR, 694 ms; TE, 9.6 ms; ETL, 4; slice thickness, 3 mm; slice gap, 0 mm; matrix size, 320 × 256; scan time, 3 min 39 s; (2) T2‐weighted image; TR, 9592 ms; TE, 102 ms; matrix size, 416 × 256; ETL, 20; scan time, 4 min 21 s.

## 3 RESULTS

### 3.1 MR image with the designed RF coil on a 3.0 T MR system and safety evaluation

Figure 2a shows MR images of the loading phantom using the designed RF coil with four temperature probes. During an MR examination that was performed for about 30‐min, the temperature was maintained within 1°C of the temperature difference (specific absorption rate (SAR, 2.5871 W/kg) measured with the FSE sequence (ETL, 32; NEX, 16)) between before and after the examination (Figure 2b). IR temperature imaging showed minimal changes but within acceptable limits according to the International Electrotechnical Commission (IEC) standard (Figure 2c).

### 3.2 Comparison with the standard 3.0 T MR‐dedicated breast coil

**FIGURE 3:**
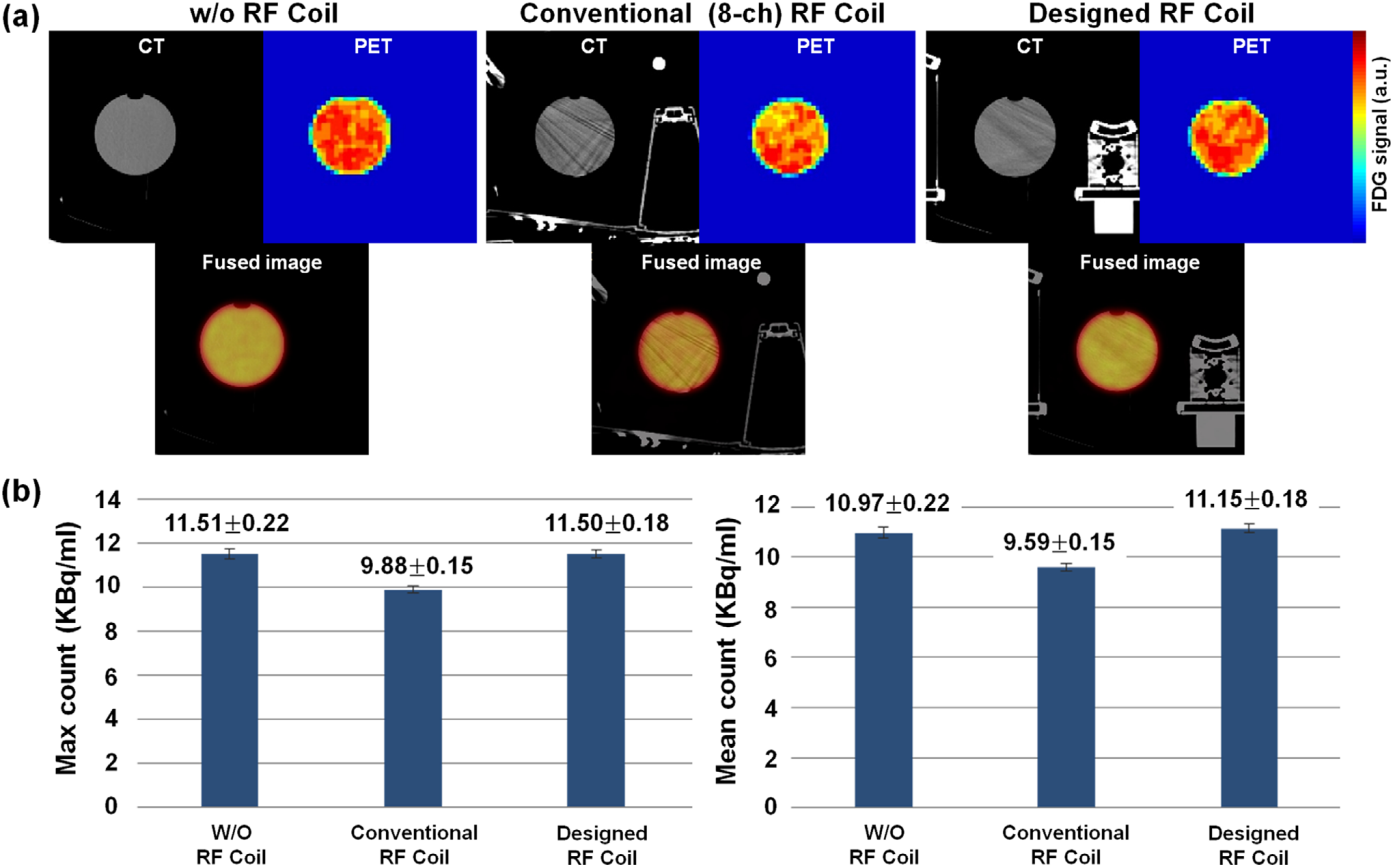
Computed tomography (CT) and positron emission tomography (PET) images of each coil. (a) CT images of the plastic bottle with normal saline using the without (w/o) radiofrequency (RF) coil, the conventional RF coil, and the designed RF coil. Beam‐hardening artifacts are seen prominently in the image produced with the conventional coil. The photon density count map of PET scan with fluorodeoxyglucose (FDG) shows more prominent artifacts for the conventional breast RF coil compared with the designed coil. (b) Photon density count map of PET and the max and mean counts. Max count of the standard conventional RF coil is low (middle column). Max count of the designed coil is similar to that of w/o RF coil. The conventional standard RF coil shows a high standard deviation, which corresponds to heterogeneous photon density count map

**FIGURE 4:**
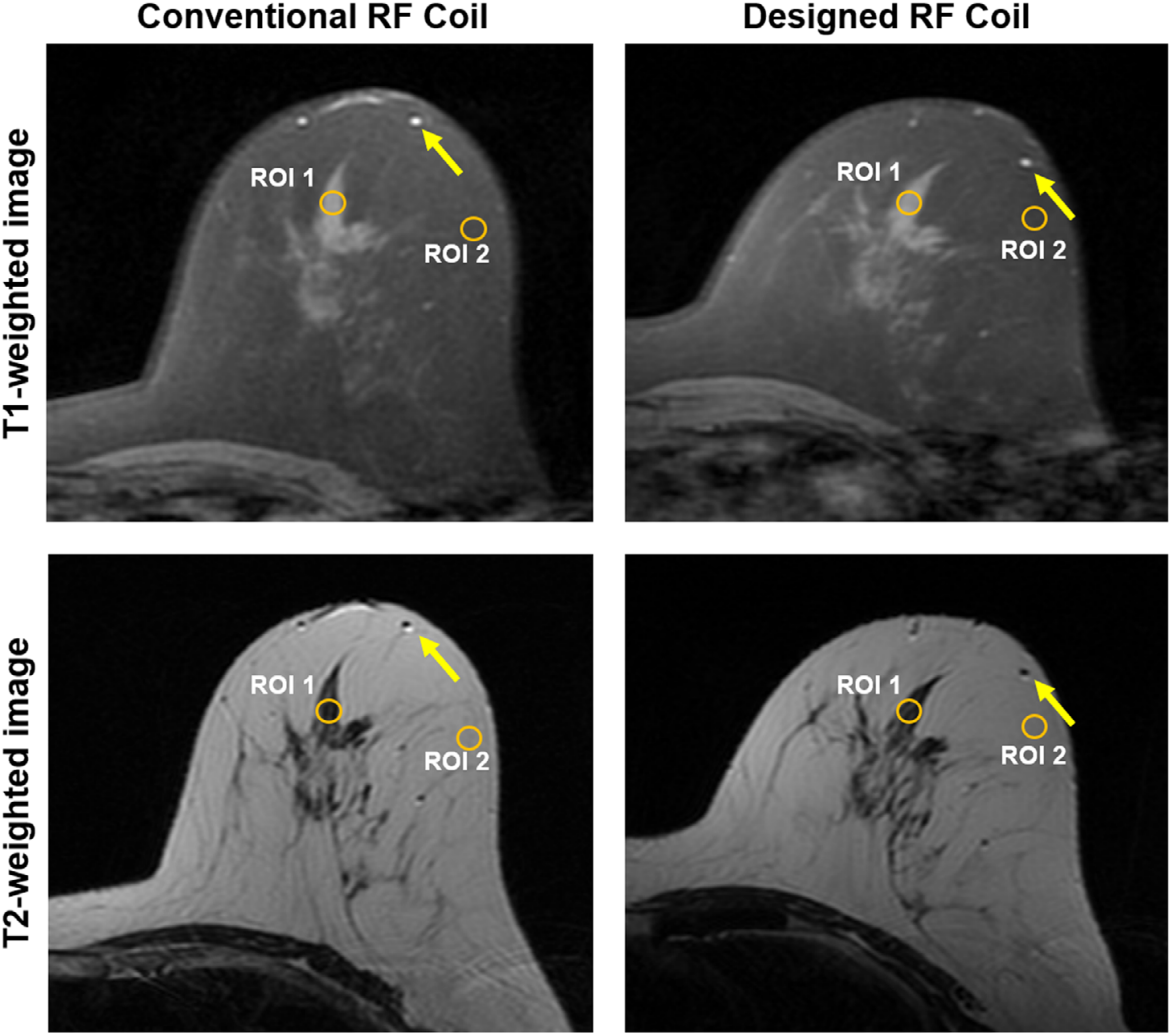
Clinical breast magnetic resonance (MR) images obtained in a volunteer using the designed radiofrequency (RF) coil and conventional coil with T1‐ and T2‐weighted images (upper column and lower column). Arrows (yellow color) indicate vascular conspicuity. A circular region of interest (ROI) (orange color) was selected to measure the image quality

Regarding image quality during the PET‐CT scan, which used the same window setting as the CT scan, we were able to see more streak signs that suggested beam hardening with conventional coil compared with the designed coil, especially in the plastic bottle (Figure 3a). The average HU ± standard deviation in center ROI was 0.41 ± 3.72 HU for the center slice of the phantom with or w/o coil, ‐4.51 ± 13.54 HU with the designed coil, and 2.38 ± 29.36 HU with the standard conventional coil. Each peripheral ROI of 12 o'clock, 3 o'clock, 6 o'clock, and 9 o'clock were 4 ± 4 HU, 0.59 ± 3.3 HU, 4.8 ± 4.6 HU, 6.2 ± 4.4 HU; ‐7.1 ± 8.3 HU, ‐22.9 ± 23.7 HU, ‐22 ± 22.7 HU, 2.7 ± 9.64 HU; and ‐1.9 ± 14 HU, 3.1 ± 22.1 HU, 11.4 ± 35.1 HU, ‐7.9 ± 22 HU, respectively. The standard deviation with the designed coil was lower than that with the conventional coil. The photon density count map of PET with FDG in the plastic bottle showed more prominent artifacts for the standard conventional coil and decreased SUVs compared with the designed coil (Figure 3a). The photon density count map with the designed coil showed a homogeneous density count equal to that with or w/o coil. The max count of the conventional coil was low (9.88 ± 0.15). The max count of the designed coil (11.50 ± 0.18) was similar to that of the w/o coil (11.51 ± 0.22). The conventional coil showed a high standard deviation (10.97 ± 0.65), which corresponded to the heterogeneous photon density count map (Figure 3b). From the designed coil, the ratio between the unloaded (*Q* value, 208.5) and loaded (*Q* value, 38.1) measurements was estimated to be 5.47. However, %PIU was 89.22%–91.27% for the designed coil and 94.63%–94.77% for the conventional coil. Figure 4 shows clearly delineated fine morphological features such as a 2‐mm‐wide blood vessel and parenchymal contour on both the standard conventional coil and designed coil with T1‐ and T2‐weighted images. The differences in SNR between the conventional and the designed coil was 175.2 versus 207.7 in ROIs of parenchymal and fat tissue regions.

## 4 DISCUSSION

Although breast MR image is the most widely used imaging modality for the initial staging of breast cancer with its high sensitivity, FDG‐PET provides functional images of metabolism with high specificity and is useful when staging locally advanced breast cancer and restaging breast cancer with recurrence. Therefore, PET‐MR hybrid imaging is a potentially promising imaging modality for the breast. However, differences in patient positioning between FDG‐PET and breast MR image and the need for a dedicated breast coil have impeded the application of PET‐MR hybrid images in breast imaging. A dedicated breast coil is crucial for breast MR, as it allows patients to maintain the more comfortable prone position along with better separation of deep breast tissue from the chest wall. Recently, some studies have suggested that the prone position can also be more effective for PET scanning in breast cancer for the same reasons as MR image because better separation of deep breast tissue, axillary, and chest wall structures might also be possible with the prone position in PET.^17^, ^18^ Therefore, PET‐MR hybrid imaging should be performed under a prone position. With the integration of a breast coil into the PET‐MR system, the attenuation of PET emission data by the RF breast coil needs to be investigated, which might be regarded as a major obstacle in the application of the PET‐MR hybrid system in breast imaging. Mainly, the metallic component of the MR coil can disturb photon transmission and image acquisition during the PET scan, which leads to decreased image quality and more image artifacts.^9^ In this study, the coil was modified to have fewer metallic components, specifically with a ceramic capacitor and plastic screws (Figure 1). For the safety evaluation of MR or PET image performance with inserted breast coil, temperature changes were monitored during scanning and confirmed as acceptable with an optic temperature probe and temperature imaging camera.

The presence of electronic components in the RF circuits with high X‐ray attenuation coefficients is common in CT imaging and yields beam‐hardening artifacts and attenuates PET emission. Therefore, the attenuation information required to reconstruct quantitative PET images is usually based on the CT scan, and several CT‐based AC techniques have been developed.^15^, ^16^, ^20^ The attenuation of PET emission data showed about 11% loss in counts due to the presence of the RF breast coil in the PET FOV.^16^ In that study, with AC techniques, the relative difference between no coil decreased to ‐4%–4% from 4%–16%, and the standard deviation also decreased from 1.2–2.7 to 1.1–2.0.^16^ This was consistent with our PET images; the maximal and mean photon density count of the designed RF coil was comparable with or w/o coil and higher than the standard conventional coil. Moreover, it was also consistent with CT images without AC (not shown). Furthermore, with the designed coil, the standard deviation of HU in the phantom was halved, compared with the standard conventional coil using the AC technique, showing how our re‐design of the coil elements can help minimize artifacts. Hardware components such as RF coils dedicated for breast imaging are not apt to be visualized on MR images.^21^ Therefore, the scatter and attenuation change from the RF coils cannot be obtained from the MR imaging data achieved in the PET‐MR system. Accordingly, several studies have tried to correct attenuation using predefined attenuation maps or a CT‐based coil template.^14^, ^22^ This study suggested that the deletion and rearrangement of metallic hardware components could alleviate the burdens of the AC software program.

For the MR images in this study, we could not compare the imaging quality of the phantom with and w/o the coil, as we cannot evaluate MR images without the coil. The SNR was higher for the designed RF coil than the standard conventional coil, while the %PIU was slightly lower for the designed coil than for the standard conventional coil, which can be affected by the number of coils and the distance from the coil. We can insist that the clinical and non‐clinical (phantom) images scanned with the designed coil have a higher SNR than other coils, which can be explained by the narrow width of the coil along with the short distance between the coil and the object of the designed coil. And our results show that the designed coil has similar or higher image quality for clinical MR images compared to standard conventional coil when considering fine morphological features. The application of multiple coil loops should improve image homogeneity, which is consistent with our results. Overall, this study demonstrated the feasibility of the designed coil for PET‐MR imaging, with comparable image quality to the standard conventional coil and fewer artifacts on the PET‐CT scan. Even though post‐processing the PET scan with AC can improve image quality, a decrease in the sources producing artifacts is very important for the future development of PET‐MR hybrid imaging.

There are some limitations in this study. The designed coil had a different number of channels from the standard conventional coil and this needs to be verified before future clinical application. A breast coil with a higher channel compatible to the PET‐MR hybrid system should be the next theme of research. In addition, clinical images did not include patients with breast cancer and the MR‐PET system needs to be verified with enhanced images of breast cancer or lesions. However, this study highlights the need to consider metallic artifacts when developing a PET‐MR hybrid system.

## 5 CONCLUSIONS

In this study, we developed a preliminary RF coil design with Helmholtz loops of transmit–receiver coils for breast MR image that can be used in a PET‐MR system. PET and MR image experiments using various phantoms showed that the proposed RF coil could provide comparable image quality for SNR and HU and result in fewer artifacts on the PET scan than the standard dedicated breast MR coil. These preliminary findings warrant further investigation and future modifications of our design will help develop a less metallic breast MR coil that is suitable for use in an MR‐PET hybrid system.

## AUTHOR CONTRIBUTIONS

Kyu‐Ho Song, Young Han Lee, Jaemoon Yang, and Min Jung Kim wrote the main manuscript. Kyu‐Ho Song, Young Han Lee, Won Jun Kang, Keum Sil Lee, Dan Heo, and Min Jung Kim collected the data and performed the data analyses. Dan Heo, Eun‐Kyung Kim, Bo‐Young Choe, and Jin‐Suck Suh reviewed the manuscript.

## CONFLICT OF INTEREST

The authors declare no conflict of interest.
